# We see what we are trained to see, or must we? Some personal lessons from a brush with kuru research

**DOI:** 10.1098/rstb.2008.4009

**Published:** 2008-11-27

**Authors:** Cyril C. Curtain

**Affiliations:** School of Pathology, University of MelbourneVictoria 3010, Australia; The Mental Health Research InstituteParkville, Victoria 3052, Australia

It began in 1957 when I received via a colleague a batch of sera from kuru patients and some controls from Carleton Gajdusek. I was immensely excited, as was Carleton, when I found a quite remarkable elevation of some α and β globulin components in the kuru sera that seemed to be absent from the controls. However, our excitement cooled in the face of more data that indicated that the sera in question had come from very sick, malnourished people suffering from major intercurrent infections, decubitus ulcers and the like. That is, we were observing a reactive hyperglobulinaemia, an epiphenomenon rather than a finding central to kuru. My overreaction to the initial finding was almost certainly due to the fact that at the time I was very interested in the paraproteinaemias of myeloma and other malignancies of the immune system and was ready to put down our kuru findings to some unique dysproteinaemia. So, the first great lesson that dipping into kuru research taught me was that we see what we are trained to see.

In 1953, the anthropologists Ronald and Catherine Berndt thought that the disease reflected a hysterical reaction of the Fore people to existential threats to their lifestyle by the advent of Europeans in the Eastern Highlands. So, this was another example of scholars seeing what they were trained to see.

However, the kuru genetic hypothesis was the prime illustration of the dangers posed by a firmly held scientific viewpoint when held by an influential group. The first genealogies collected in the field showed that the disease had a strong family association and, in their incomplete state, the data could reasonably be held to point to the existence of a *Ku* gene, possibly with its expression modified by a recently introduced environmental factor. In the early years, nearly all who thought about the disease conceded that genetics might play some part.

Prof. J. H. Bennett, the chief protagonist of the genetic hypothesis, held the chair of genetics at Adelaide University. He was a fine classical geneticist and his erstwhile Cambridge mentor, Sir Roland Fisher FRS, was at the time in Adelaide as guest of the CSIRO (Commonwealth Scientific and Industrial Research Organization). Backed by H. N. Robson, Prof. of Medicine at Adelaide, they became known as the Adelaide Group, which went on to enjoy powerful leverage with sections of the Australian political and medical establishments. Politically, the Group's involvement was a demonstration that we took our trusteeship of the Territory of Papua and New Guinea seriously and were not prepared to leave the field to Carleton who was seen as foreign invader. This was an important consideration when colonial administrations of all types were being heavily criticized. The anglophile sensitivities of many in the medical establishment had been outraged that an American was playing on their pitch and they saw the existence of the Adelaide Group as an important expression of our concern for the welfare of the Fore people. None of this might have mattered much had it stopped at being a reflection of healthy academic chauvinism, spiced with a little cold war *realpolitik*, since Carleton had the ability and means to carry out a comprehensive study of kuru by himself, while the Adelaide Group could have busied itself verifying, or falsifying, the genetic hypothesis. Unfortunately, the chauvinism became decidedly unhealthy with proposals to exclude Carleton from the field, which would have given the collection of genealogies a head start. Such a situation would have been very satisfying to those who argued that Australia could solve the kuru problem from its own resources, an argument based on our undoubted strengths in genetics. While eventually the genetic hypothesis was falsified, its monopoly of the field, even for a season, could have disrupted many of Carleton's collaborative arrangements with unpredictable results. Just as seriously, the genetic hypothesis was so strongly held that proposals arose to ‘quarantine’ the Fore people so that the hypothetical *Ku* would not spread elsewhere in Papua New Guinea; even eugenic measures such as sterilization were contemplated.

Compared with the other public health problems facing the country, to the Papua New Guinea authorities kuru was a minor diversion that threatened to blow up into an international incident. Faced with this situation, the administration and its advisers sought independent opinions that queried the strengths of the arguments for a genetic quarantine. Interestingly, very few of those who advised against eugenic measures did so on moral grounds but were more concerned with their impact on public opinion and the practicality of enforcement. About the same time, opposition to Carleton's early return to the field was dropped. Again, the decision was based on pragmatism rather than principle. Carleton had the full support of the National Institutes of Health of Australia's ‘great and powerful friend’ and it was becoming clear, even to the sceptics, that his energetically pursued multi-pronged approach offered the best chance of understanding this baffling disease. From then on, the Papua New Guinea Department of Public Health gave him every assistance, making a major local contribution to solving the problem in its own right.

The final lessons that I learnt from those early kuru years were that tightly held hypotheses and public policy were a dangerous mix and that bureaucratic decision makers and their advisers frequently favour expediency over principle. Although in the case of kuru the issues were simple enough for those of goodwill to negotiate a positive solution, in more complex situations such an approach has frequently ended in disaster. These lessons have stayed with me over a varied career that included some studies, independent of kuru, in Papua New Guinea and periods as a research programme manager in various settings in CSIRO.

